# Diverse Cell Populations Involved in Regeneration of Renal Tubular Epithelium following Acute Kidney Injury

**DOI:** 10.1155/2015/964849

**Published:** 2015-05-18

**Authors:** Akito Maeshima, Shunsuke Takahashi, Masao Nakasatomi, Yoshihisa Nojima

**Affiliations:** Department of Medicine and Clinical Science, Gunma University Graduate School of Medicine, 3-39-15 Showa, Maebashi 371-8511, Japan

## Abstract

Renal tubular epithelium has the capacity to regenerate, repair, and reepithelialize in response to a variety of insults. Previous studies with several kidney injury models demonstrated that various growth factors, transcription factors, and extracellular matrices are involved in this process. Surviving tubular cells actively proliferate, migrate, and differentiate in the kidney regeneration process after injury, and some cells express putative stem cell markers or possess stem cell properties. Using fate mapping techniques, bone marrow-derived cells and endothelial progenitor cells have been shown to transdifferentiate into tubular components in vivo or ex vivo. Similarly, it has been demonstrated that, during tubular cell regeneration, several inflammatory cell populations migrate, assemble around tubular cells, and interact with tubular cells during the repair of tubular epithelium. In this review, we describe recent advances in understanding the regeneration mechanisms of renal tubules, particularly the characteristics of various cell populations contributing to tubular regeneration, and highlight the targets for the development of regenerative medicine for treating kidney diseases in humans.

## 1. Introduction

Renal tubules express several types of transporter that are involved in renal reabsorption and secretion, as well as ion channels for the maintenance of body fluid balance. These cells comprise polarized mature epithelial cells with the capacity to regenerate following acute kidney injury. After the insult occurs, surviving tubular cells rapidly lose epithelial cell properties and acquire a more mesenchymal phenotype. The dedifferentiated cells migrate into the regions where cell necrosis, apoptosis, or detachment has resulted in denudation of the tubular basement membrane. They proliferate and eventually differentiate into mature epithelial cells with polarized lumen, completing the repair process [1].

**Figure 1:**
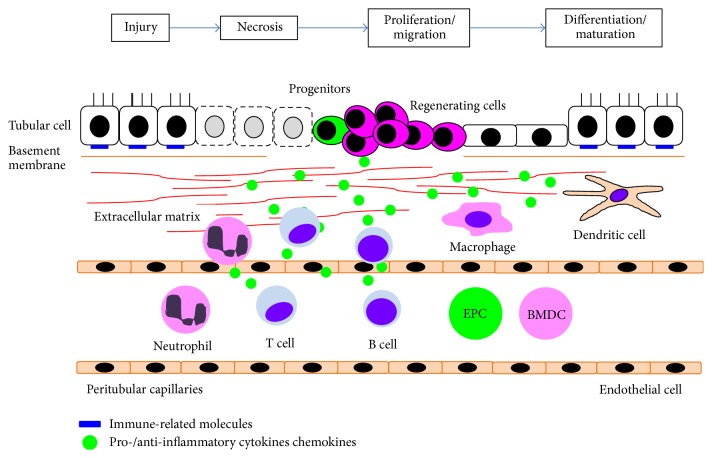
Diverse cell populations involved in tubular regeneration after injury.

The process of restoration and maturation of damaged epithelium after renal injury has many parallels with the developmental process during kidney organogenesis. Soluble factors involved in kidney development have been identified by gene targeting techniques, in vitro tubulogenesis models, and organ culture systems, and most of these also have been demonstrated to regulate kidney recovery as potential renotrophic factors [2]. These factors have been shown to be epithelial cell mitogens in vitro and to induce tubular cell proliferation after injury when exogenously administered. With recent fate mapping techniques that facilitate cell lineage tracing, various cell populations or cell-cell interactions have been revealed to be intricately involved in tubular regeneration after acute kidney injury (Figure 1).

In this review, we highlight recent advances regarding the regeneration mechanisms of renal tubules after injury, particularly focusing on possible cell populations and their interactions, which contribute to the repair process of renal tubules after injury.

## 2. Regeneration Process of Renal Tubules after Injury

Renal tubular epithelium has a huge capacity for regeneration after injury. During the repair process, surviving tubular cells actively proliferate and differentiate into mature tubular cells to reconstruct their functional structures. Sophisticated lineage tracing studies have demonstrated that it is unlikely that extrarenal cells enter the tubule and differentiate into epithelial cells in mice. It is more likely that tubule recovery is controlled by a number of intratubular cells with a substantial regenerative capacity [3, 4].

### 2.1. Potential Progenitor Cells Engaged in Tubular Repair

Despite the structural complexity of the adult kidney, attempts to identify cell populations contributing to the regenerative process have been based on the broad principles of stem cell biology. To conserve growth potential and prevent genetic injury during mitosis, stem cells cycle slowly and are recruited only as demanded by tissue turnover. To identify slow-cycling stem cells, a pulse label of 5-bromo-2-deoxyuridine (BrdU), followed by a chase period, is commonly used, allowing the detection of slow-cycling label-retaining cells (LRCs). LRCs have been identified in renal tubules of normal rat kidneys, and regenerating cells during tubular repair are essentially derived from LRCs [5–7]. The number of these LRCs declines with age, resulting in reduced regenerative capacity after injury in the aging kidney [8]. Other groups also found LRCs in tubules [9, 10], papilla [11], and renal capsules [12]. A previous study demonstrated that there is a unique cell population in rat kidneys that self-renews for more than 200 population doublings, without evidence of senescence. These cells were able to differentiate into renal tubules when injected intra-arterially after renal ischemia [13]. Another report revealed that a promising cell line derived from the S3 segment of the proximal tubules could be maintained for long periods without transformation and that the cells were partly replaced in injured tubules when engrafted to the kidney after renal ischemia [14]. These results suggest the presence of a tubular progenitor cell population with high proliferation capacity. Fate mapping studies applying targeting strategies with progenitor-specific labeling and clonal analysis tools will enable us to examine which cell population predominantly contributes to tubular regeneration after kidney injury.

In human, a resident population with progenitor characteristics was identified along the nephron [15]. Stem cell marker CD133-positive cells were localized in the Bowman's capsule of the glomeruli, in the proximal tubules as well as in the inner medullary papilla region [16, 17]. The embryonic renal markers and mesenchymal stem cell markers were found to be expressed in these cells. The number of cortical CD133-positive tubular cells increased in patients with acute renal injury [18]. These cells might be able to obtain progenitor property after damage. It has been also demonstrated that CD133/CD24 double-positive renal stem/progenitor cells secreted chemoattractants, such as MCP-1 or IL-8, that can induce recruitment or mobilization of hematopoietic or mesenchymal stem cells [19, 20]. Pharmacological manipulation of renal progenitors is one of the promising strategies to enhance intrinsic capacity of tubular regeneration after injury.

### 2.2. Immune-Related Molecules Expressed by Tubular Cells

Tubular epithelial cells play an important role in the inflammatory process after acute kidney injury. Recent studies have highlighted the importance of several tubular cell surface proteins in mediating the interaction between tubular cells and inflammation during tubular repair.

Renal tubular epithelium constitutively expresses toll-like receptors (TLRs), a family of pattern recognition receptors that detect motifs of pathogens and host material released during injury. TLRs mediate signal transduction pathways that control the expression of proinflammatory cytokines and chemokines. TLR4 is upregulated in tubular epithelial cells after renal ischemia, while TLR4 deficiency decreases the renal ischemic injury-induced production of proinflammatory cytokines and chemokines and inhibits macrophage and neutrophil accumulation [21]. Lack of TLR2 expression in kidney parenchymal cells also inhibits renal ischemic injury. Production of proinflammatory cytokine is reduced in TLR2-deficient mouse kidneys when compared with wild-type kidneys [22]. In vitro data have demonstrated that stimulation with lipopolysaccharide upregulates the expression of TLR2, TLR3, and TLR4 and secretion of C-C chemokines, in cultured mouse tubular cells, thus suggesting the involvement of tubular TLRs in mediating interstitial leukocyte infiltration and tubular injury [23].

The complement system has been shown to mediate renal ischemia-reperfusion injury. Normally, proximal tubular epithelial cells express Crry, a complement inhibitor, on the basolateral membrane. After renal ischemia, Crry production decreases, thus allowing the deposition of C3 on the tubular epithelium and stimulation of proinflammatory CXC chemokine production. These chemokines attract neutrophils and macrophages to the injured kidney [24]. In support of a protective role for proximal tubular Crry expression, Crry-deficient mice are more susceptible to renal ischemic injury [25]. Activation of C5a receptor (C5aR) is known to induce the recruitment of neutrophils and macrophages and activates these to produce cytokines, chemokines, and adhesion molecules. After renal ischemia, C5aR expression was upregulated in tubular epithelial cells. Blockade of the C5aR pathway with a specific C5aR antagonist abolished upregulation of CXC chemokines and significantly reduced renal damage [26].

Suppressor of cytokine signaling 3 (SOCS-3), a key intracellular negative regulator of several signaling pathways, has been found to be strongly expressed in renal proximal tubules after acute kidney injury. Conditional proximal tubular knockout mice showed accelerated tubule recovery after renal injury, which is associated with increased numbers of infiltrating anti-inflammatory M2 macrophages during the repair phase. These results suggest that upregulated expression of SOCS-3 in damaged proximal tubules inhibits the regeneration process and modulates macrophage phenotype [27].

## 3. Recruitment of Inflammatory Cells during Tubular Regeneration

Repeated or persistent injury results in cell death and limited tubule regeneration. Recruitment of leukocytes is recognized as a major mediator of tubular cell injury. The initial stages of inflammation are characterized by migration of leukocytes to the activated vascular endothelium, followed by transmigration into the interstitium. During the injury phase, tubular epithelial cells express proinflammatory cytokines and chemokines, which recruit neutrophils, M1 macrophages, and various proinflammatory lymphocyte subsets [28].

### 3.1. Neutrophils

Neutrophils rapidly respond to injury, and adherence of neutrophils to the vascular endothelium is a crucial process in the initiation of damage to ischemic tissues [21]. Neutrophil accumulation is one of the hallmarks of renal ischemic injury, and depletion of neutrophils inhibits acute kidney injury [29]. However, blockade of neutrophil function or neutrophil depletion provides only partial protection against renal injury, indicating that other leukocytes, such as macrophages, B cells, and T cells, also mediate acute kidney injury [30].

### 3.2. Lymphocytes

Lymphocytes are also important for tubular repair after ischemic injury. B cells migrate to the ischemic kidney and differentiate into plasma cells, which limit tubule regeneration after acute kidney injury. Consistent with these data, B cell-deficient mice are protected from renal ischemic injury [31, 32]. However, the adoptive transfer of purified B cells back into these mice does not repair kidney injury after ischemia. On the other hand, when compared to B cell-deficient mice without serum transfer, the transfer of serum from wild-type mice results in higher serum creatinine levels after renal ischemia, thus suggesting that the absence of a circulating factor is responsible for the protection observed in B cell-deficient mice. Other investigators have reported no protection from renal ischemic injury in RAG-1-deficient mice lacking both B cells and T cells [33].

The role of T cells in the pathogenesis of renal ischemic injury has been analyzed in different mouse models. T cells have a major role in vascular permeability, potentially through production of cytokines during ischemic injury. Gene microarray analysis has shown that production of TNF and interferon-gamma increased in CD3-positive and CD4-positive T cells from the blood and kidney after renal ischemia. In mice lacking T cells expressing CD3, CD4, and CD8, the increase in renal vascular permeability was attenuated after ischemic injury [34, 35]. In mice lacking CD4-positive and CD8-positive T cells, serum creatinine levels and renal histology decreased significantly after renal ischemia. Reconstitution of mice with CD4-positive T cells alone, but not CD8-positive T cells alone, restored kidney function after renal injury [36]. In addition, RAG-1-deficient mice lacking both B and T cells are also protected from renal ischemic injury and adoptive transfer of CD4-positive T cells from wild-type mice reconstitutes injury. Importantly, transfer of CD4-positive T cells from IFN-gamma-deficient mice failed to repair injury in this model [37], thus suggesting that IFN-gamma-producing CD4-positive T cells mediate the early phases after renal ischemic injury. Exposure of renal tubular epithelial cells to 2 h of hypoxia followed by 1 h of reoxygenation increased T cell adhesion by more than twofold. Phorbol ester treatment, which activates integrins, increased T cell adhesion. These data suggest that T lymphocytes mediate renal ischemic injury. Adhesion of infiltrating T cells to renal tubular cells is a critical step underlying postischemic tubular dysfunction [38].

Among several lymphocyte subsets with anti-inflammatory properties, regulatory T cells (T_REG_ cells) have been shown to promote tubular repair, possibly through the modulation of proinflammatory cytokine production of other T-cell subsets after acute kidney injury. Increased T_REG_ cell trafficking into the kidneys was observed after ischemic injury. Infusion of T_REG_ cells after initial injury reduced interferon-gamma production by T cell receptor (TCR) *β*+CD4+ T cells, improved renal repair, and reduced cytokine generation. In contrast, partial depletion of T_REG_ cells with an anti-CD25 antibody potentiated ischemia-induced kidney damage and resulted in more neutrophils, macrophages, and innate cytokine transcription in the injured kidney [39, 40]. Mice deficient in T_REG_ cells had a greater accumulation of inflammatory leukocytes after renal ischemia than mice containing T_REG_ cells [41].

Natural killer T (NKT) cells, a unique subset of T lymphocytes with surface receptors and functional properties shared with conventional T cells and natural killer (NK) cells, have also been shown to be involved in tubular regeneration after injury. Invariant NKT (iNKT) cells possess a conserved invariant TCR together with the NK cell marker NK1.1. Renal ischemic injury leads to an increase in activated CD4+CD69+ cells, and the number of IFN-gamma-producing iNKT cells in the kidney increases significantly after renal ischemia. Blockade of NKT cell activation with an anti-CD1d monoclonal antibody, NKT cell depletion with an anti-NK1.1 monoclonal antibody in wild-type mice, or use of iNKT cell-deficient mice inhibited the accumulation of IFN-gamma-producing neutrophils after renal injury and reduced acute kidney injury [42]. These studies suggest that neutrophil activation and infiltration is regulated by iNKT cells.

### 3.3. Macrophages

Macrophages are derived from monocytes in the blood and are named for their role in phagocytosis. In addition, macrophages produce proinflammatory cytokines that can stimulate the activity of other leukocytes. Macrophages are involved in all phases of tissue injury, including regeneration [43]. Several macrophage-derived cytokines not only have immunosuppressive effects but also enhance the repair process. Macrophages produce Notch ligands, which stimulate the proliferation of tubular epithelial cells [44]. Blockade of Notch signaling with a gamma-secretase inhibitor suppresses Notch-2-driven induction of survivin and its autocrine capacity to regenerate tubules in a model of ischemic kidney injury [45]. IL-6 produced by interstitial macrophages in renal outer medulla mediates renal ischemic injury [46].

Macrophages infiltrate the injured kidney shortly after renal ischemia and this infiltration is mediated by CX3CR1 signaling pathways [47]. Depletion of kidney and spleen macrophages, using liposomal clodronate, prior to renal ischemic injury prevented acute kidney injury, and adoptive transfer of macrophages reconstituted acute kidney injury [48]. It was recently reported that neutrophil accumulation is controlled by vascular-resident CD169-positive macrophages after renal ischemia [49].

It is thought that proinflammatory stimuli preserve M1 macrophage activity that induces tubular atrophy. Sustained secretion of TNF partially accounts for these antiregenerative effects, which potentially triggers tubular cell injury. Therefore, tubular regeneration can be observed only when M1 macrophages are deactivated [50, 51]. In contrast, macrophage colony stimulating factor-1 signaling leads to proliferation of M2 macrophages with proregenerative properties, which support the resolution of inflammation and acceleration of tubular regeneration during the recovery phase of acute kidney injury [51]. During tubular recovery after injury, M2 macrophages secrete Wnt7b, which interacts with Wnt receptors on the surviving tubular epithelial cells and accelerates tubule recovery via Wnt-*β*-catenin signaling [52].

### 3.4. Dendritic Cells

Dendritic cells, an important link between innate and adaptive immunity, are abundant in the normal mouse kidney. Upon stimulation, dendritic cells develop into a mature cell type characterized by high levels of class II major histocompatibility complex (MCH class II) and costimulatory molecules and low phagocytic capacity. Mature dendritic cells are specialized in T cell activation. However, dendritic cells are also important in the innate immune response by releasing proinflammatory factors, interacting with NKT cells via CD40-CD40L and presenting glycolipids via the CD1d molecule to activate iNKT cells.

Dendritic cells play a role in acute kidney injury. After acute kidney injury, renal dendritic cells produce the proinflammatory cytokines TNF, IL-6, C-C motif chemokine 2, and C-C motif chemokine 5, and depletion of dendritic cells before ischemia substantially reduces the levels of TNF produced by the kidney [53]. In a separate study, dendritic cells were shown to accumulate in the renal draining lymph nodes after renal ischemic injury and induce T cell proliferation in an antigen-specific manner, thus suggesting that renal dendritic cells are involved in the adaptive immune response to renal ischemic injury [54].

The activation of intrarenal dendritic cells by factors released from dying tubular cells also promotes tubule regeneration. Necrotic tubular cells release a number of intracellular molecules that act as immunostimulatory DAMPs, specifically activate TLR4 on intrarenal dendritic cells, and induce secretion of IL-22 [55]. Dendritic-cell-derived IL-22 activates the corresponding receptor and subsequent STAT3 and Erk signaling in surviving tubular cells, leading to the enhancement of tubular regeneration and recovery [55].

## 4. Role of Peritubular Capillary Endothelium in Tubular Recovery

The integrity of vascular endothelium is determined by the balance between endothelial turnover and repair. In the kidney, peritubular capillary endothelium is reported to act as a source of factors required for tubular recovery after injury [56]. However, a considerable decrease in the density of peritubular capillary is observed following acute ischemic injury indicating that, unlike renal epithelial tubular cells, the renal vascular system lacks comparable regenerative potential [57, 58]. After ischemic insult, damaged endothelial cells slough into the circulation, and replacement occurs via the induced proliferation of neighboring endothelial cells and/or by the recruitment of EPCs (endothelial progenitor cells) from the circulation. Growing evidence suggests that the bone marrow is a rich source of immature EPCs. Circulating EPCs can be recruited into vascular beds to maintain normal physiologic homeostasis/repair. Similarly, it has been reported that renal ischemia mobilizes EPCs and induces the accumulation of EPCs in the renal medulla, and transplantation of EPC-enriched cells from the medullary parenchyma affords partial renoprotection after renal ischemia, thus suggesting a role for recruited EPCs in functional rescue [59, 60]. Paracrine mechanisms of therapeutic effect of EPC are also reported. Microvesicles derived from EPC protect the kidney from ischemic acute injury by delivering their RNA content, the microRNAs cargo which converts survived resident renal cells into a more regenerative state [61].

Peritubular capillary endothelium also plays an important early role in the inflammatory response to kidney damage by promoting the accumulation of leukocytes. After renal ischemic injury, capillary endothelium is activated, leading to an increase in vascular permeability [62], which promotes recruitment of leukocytes into the kidney. In addition to changes in the integrity of the endothelial cell layer of the renal vasculature, renal ischemic injury upregulates the expression of adhesion molecules that facilitate leukocyte-endothelial cell interactions. The expression of intracellular adhesion molecule 1- (ICAM-) 1 increases in the kidney after renal ischemic injury. Mice lacking ICAM-1 are protected from renal ischemic injury [29]. Leukocyte adhesion to endothelial cells leads to inflammation and extension of cellular injury. In addition, renal endothelial cells upregulate the expression of CX3CL1 (fractalkine), a ligand for the CX3CR1 receptor expressed on macrophages that mediates macrophage recruitment in the inflamed kidney, and pretreatment with a neutralizing CX3CR1 monoclonal antibody reduces the severity of acute kidney injury [47].

## 5. Recruitment of Bone Marrow-Derived Cells (BMDCs) during Tubular Regeneration

Similar to the results observed in other organs, BMDCs appear in the kidney in response to renal injury. BMDCs significantly contribute to the regeneration of the renal tubular epithelium, differentiate into renal tubules [63–65], or promote proliferation of both endothelial and epithelial cells after injury [66]. Based on these data, cell therapy with BMDCs has been extensively examined and reported to be effective. In light of their ease of accessibility, BMDCs are strong candidates as a cell source in stem cell therapy. Stem cell factor and granulocyte colony-stimulating factor (G-CSF) induce hematopoietic stem cells (HSCs) homing to the injured kidney, leading to significant enhancement of the functional recovery of the kidney [67, 68]. In contrast to the reports above, boosting of peripheral stem cell numbers was found to be associated with increased severity of renal failure and mortality. High numbers of activated granulocytes appeared to obscure the potential renoprotective effects of HSC [69]. There are several reports against the potential of BMDCs to transdifferentiate into tubular cells after injury. Transgenic mice that express GFP in BMDCs [70], in mature renal tubular epithelial cells [71], or in all mesenchyme-derived renal epithelial cells [72] revealed that, while BMDC recruitment occurs, tubular recovery after renal ischemia is predominantly elicited via proliferation of endogenous renal tubular cells.

Mesenchymal stem cells (MSCs) derived from bone marrow also have been reported to enhance the intrinsic tubular recovery in several acute kidney injury models in a paracrine manner. Treatment with MSCs promoted proximal tubular cell proliferation, reduced apoptosis, and preserved microvascular integrity, leading to the amelioration of renal tissue oxygenation [73–76]. It is considered that MSCs interact with resident cells in endocrine and paracrine manners through the release of growth factors, cytokines, or extracellular vesicles [77, 78]. MSC-derived conditioned medium, which contains renotrophic factors and anti-inflammatory factors, was shown to be able to enhance kidney repair after injury [77, 79, 80]. BMDCs including MSCs are now considered to contribute to the regenerative process by producing protective and regenerative factors, rather than by differentiating to directly replace damaged cells.

## 6. Conclusions

Recently, many new concepts in the regeneration process of renal tubules after acute kidney injury have emerged. Growing evidence suggests that the immune system supports the regeneration process of the kidney after injury. Using gene targeting techniques or fate mapping analysis, the involvement of diverse cell populations including tubular cells, inflammatory cells, resident renal endothelial cells, endothelial progenitor cells, and bone marrow-derived cells has been clarified. Critical roles for neutrophils, lymphocytes, and macrophages have been established in mouse models of acute kidney injury. In addition, several studies have reported that complement, TLRs, and numerous cytokines and chemokines are clearly involved in amplifying the immune response to kidney injury. However, the complex interplay between tubular cells and neighboring cells in renal ischemic injury is not yet fully understood. Termination of renal inflammation will enable tubular regeneration by inhibiting tubular cell necrosis or by driving a phenotype switch from proinflammatory to anti-inflammatory immune cells. In this context, specific and selective anti-inflammatory drugs will be required to suppress systemic and local causes of renal inflammation to promote regeneration in patients with kidney diseases. Specific activation of surviving tubular epithelial cells with regenerative capacity also represents an attractive approach to enable sufficient reepithelialization and nephron survival after renal injury. These new concepts will provide important clues for identifying new targets for the development of clinically relevant treatment strategies for acute kidney injury.
